# Modeling and Mapping of Atmospheric Mercury Deposition in Adirondack Park, New York

**DOI:** 10.1371/journal.pone.0059322

**Published:** 2013-03-25

**Authors:** Xue Yu, Charles T. Driscoll, Jiaoyan Huang, Thomas M. Holsen, Bradley D. Blackwell

**Affiliations:** 1 Department of Civil and Environmental Engineering, Syracuse Univeresity, Syracuse, New York, United States of America; 2 Department of Natural Resources and Environmental Sciences, University of Nevada-Reno, Reno, Nevada, United States of America; 3 Department of Civil and Environmental Engineering, Clarkson University, Potsdam, New York, United States of America; University of Illinois at Chicago, United States of America

## Abstract

The Adirondacks of New York State, USA is a region that is sensitive to atmospheric mercury (Hg) deposition. In this study, we estimated atmospheric Hg deposition to the Adirondacks using a new scheme that combined numerical modeling and limited experimental data. The majority of the land cover in the Adirondacks is forested with 47% of the total area deciduous, 20% coniferous and 10% mixed. We used litterfall plus throughfall deposition as the total atmospheric Hg deposition to coniferous and deciduous forests during the leaf-on period, and wet Hg deposition plus modeled atmospheric dry Hg deposition as the total Hg deposition to the deciduous forest during the leaf-off period and for the non-forested areas year-around. To estimate atmospheric dry Hg deposition we used the Big Leaf model. The average atmospheric Hg deposition to the Adirondacks was estimated as 17.4 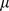
g m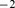 yr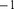 with a range of −3.7–46.0 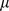
g m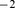 yr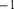. Atmospheric Hg dry deposition (370 kg yr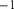) was found to be more important than wet deposition (210 kg yr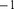) to the entire Adirondacks (2.4 million ha). The spatial pattern showed a large variation in atmospheric Hg deposition with scattered areas in the eastern Adirondacks having total Hg deposition greater than 30 *μ*g m^−2^ yr^−1^, while the southwestern and the northern areas received Hg deposition ranging from 25–30 *μ*g m^−2^ yr^−1^.

## Introduction

Ecosystems of the Adirondack Park in New York State, USA, have been substantially affected by mercury (Hg) contamination and the region is considered to be a “biological Hg hotspot” [1]–[3]. There are limited direct anthropogenic Hg emission sources within or near the Park, and atmospheric deposition is the predominant Hg input. As a result it is important to quantify the spatial pattern of atmospheric Hg deposition to evaluate the magnitude of Hg inputs as well as the factors driving landscape variations of Hg contamination in the terrestrial and aquatic ecosystems [4].

Atmospheric Hg occurs largely in three operationally defined forms: gaseous elemental Hg (GEM; 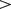
95% of the total mass), gaseous oxidized Hg (GOM), and particulate bound Hg (PBM) [5]–[7]. Atmospheric Hg input to the Earth's surface is via wet and dry deposition [6], [8]. Wet Hg deposition is well monitored by Mercury Deposition Network (MDN) at 112 currently active sites as part of the National Atmospheric Deposition Program (NADP) sites in North America [9]. However, there are limited measurements of the spatial variations of atmospheric Hg concentrations and Hg dry deposition [10], [11]. Therefore numerical modeling is often used to estimate atmospheric Hg deposition. Existing atmospheric deposition models include both global/continental scale [12]–[16], and regional scale models [17]–[25]. However, no study has systematically estimated atmospheric Hg deposition to a relatively small but ecologically important region like the Adirondacks.

Atmospheric models, either Lagrangian or Eulerian, often use a box scheme or “response” approach, to estimate the deposition flux in a defined domain. This approach is generally based on anthropogenic Hg emission inventories and/or modeled natural Hg emissions, assumes some initial atmospheric Hg concentration, and simulates the transformations and transport of atmospheric Hg forms based on certain driving parameters (physico-chemical reaction constants of atmospheric Hg, meteorological conditions, and land surfaces)[12]–[25]. The deposition flux is calculated as the product of deposition velocity (V_d_) and atmospheric Hg concentrations [26]. While these models can provide useful results to characterize Hg fluxes due to air-surface exchange, their limitations include: (1) inaccuracies/uncertainty in documenting/estimating Hg emissions, especially non-point anthropogenic emissions and natural emissions [27]; (2) gaps in understanding Hg speciation and physico-chemical reactions in the atmosphere, such as those involving particles and cloud droplets [28]–[30]; (3) uncertainties in the physico-chemical mechanisms of Hg exchange between the atmosphere and Earth surfaces [31], [32]; and (4) the use of a relatively coarse grid size (e.g. 12 km: [18], [33]; or 36 km: [34]) that is not sufficient to reflect important spatial variations of atmospheric Hg deposition to the local environment and particularly under complex topography like occurs in the Adirondacks. In this study, we developed a modified scheme, or “surface receptor” model to estimate atmospheric Hg deposition to the Adirondacks based on both measured atmospheric Hg concentrations; wet, litterfall and throughfall Hg deposition; and numerical modeling of atmospheric Hg dry deposition velocities.

## Methods

### Site Description

**Figure 1:**
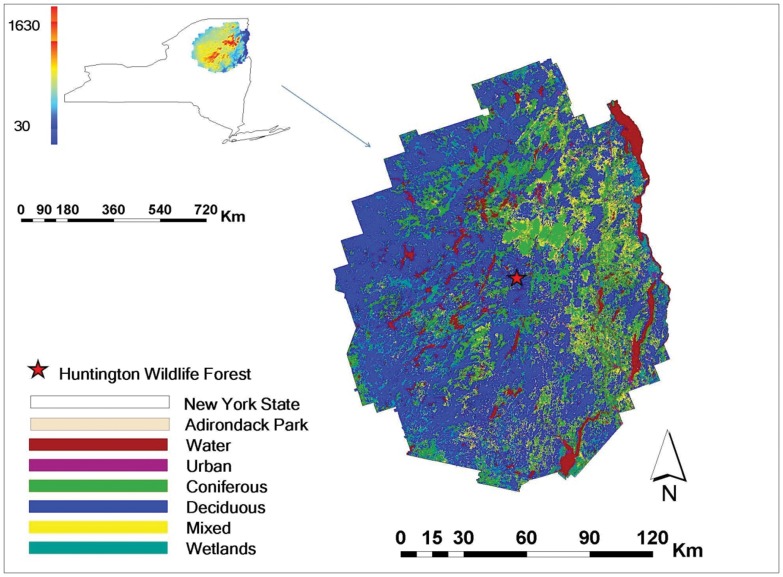
Location of Adirondack Park in New York State and the elevation (unit: m) distribution in the Park (upper panel), and the land cover distribution pattern of the Park (lower panel). The location of the Huntington Wildlife Forest is represented by the star. In this paper, we used the same color scheme to represent the LUCs in all the figures.

The modeling domain of this study is the Adirondack Park of New York State (43°00'–44°55'N, 73°15'–75°20'W), which covers an area of 2.4 million ha with a unique landscape of mountains, wetlands and lakes, and northern hardwood, boreal and alpine tundra vegetation that is sensitive to Hg deposition [35]. The mean elevation is 460 m with a range of 30–1630 m (Figure 1). Forest is the largest land use category (LUC), which accounts for 77% of the total land area with 47% as deciduous forest, 20% as coniferous forest, and 10% as mixed forest. The remaining LUCs are mainly woody wetlands (11%), and open water (6%). The dominant tree species in the deciduous forest are sugar maple (*Acer saccharum*), American beech (*Fagus grandifolia Ehrh.*), and yellow birch (*Betula alleghaniensis*), while coniferous forest is dominated by red spruce (*Picea rubens*) and balsam fir (*Abies balsamea (L.)P.Mill*) [36].

**Figure 2:**
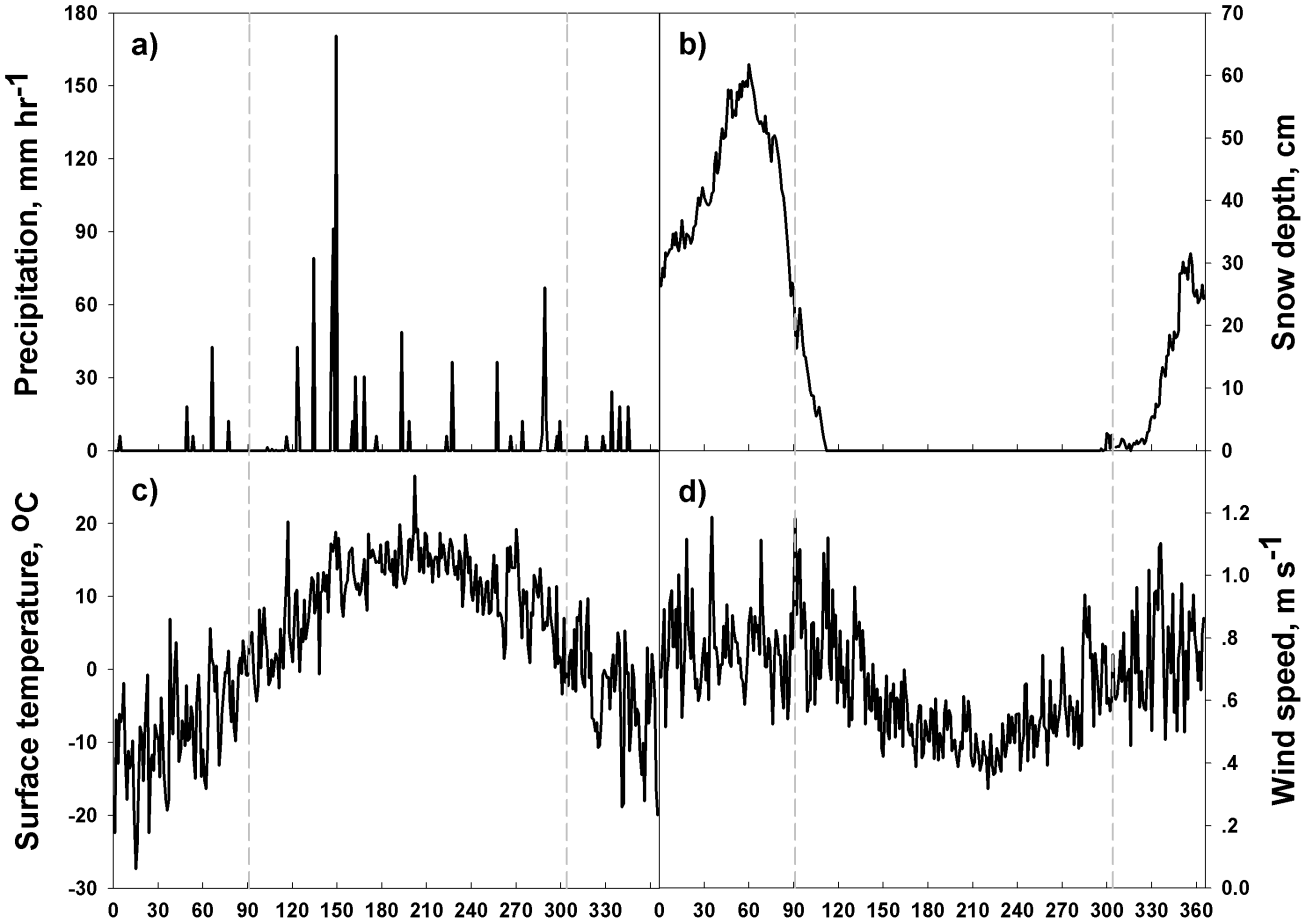
Daily average seasonal meteorological conditions at Huntington Wildlife Forest for the period 2009–2011 measured by CASTNET. Temperature and wind speed exhibited much diurnal variations. However their annual patterns followed the same patterns presented here. Precipitation and snow were mainly event-based. The area between the gray vertical dashed lines represent the leaf-on period.

The Huntington Wildlife Forest (HF) in Newcomb (43.97°N, 74.22°W; elevation: 500 m) is an intensive, long-term ecosystem study area, and has an MDN site (NY 20) and a Clean Air Status and Trends Network (CASTNET, HWF187) site (US EPA; http://epa.gov/castnet/javaweb/index.html) which monitors meteorological conditions on an hourly basis. We used these data from Jan 2009–Dec 2011 (Figure 2) and defined the leaf-on period as Apr–Oct, and the rest of the year as the leaf-off period. The annual temperature (mean 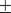 stdev) was 7.4
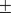
10.8°C, with 12.4
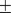
7.2°C during the leaf-on period and −5.0
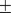
7.7°C during the leaf-off period. Surface temperature was significantly correlated with solar radiation (r = 0.81, p
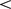
0.0001). The annual precipitation was 1070
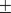
6300 mm, with greater precipitation in the late spring. The average annual wind speed was 0.64
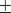
0.53 m s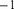, with a statistically greater wind speed during the leaf-off period (0.75
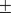
0.56 m s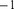, n = 9148) than the leaf-on period (0.55
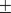
0.51 m s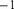, n = 15206; p
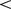
0.0001, GLM Tukey's method). The general wind direction was from the southwest, which has implications for the transport of Hg from important source areas in the Midwest [37]. Note local wind direction may be affected by the presence of trees and changes in elevation.

### Experimental and Data Analysis Methods

We measured atmospheric Hg concentrations from Jan 2009–Dec 2011 on a 3-hour basis using a Tekran Model 2537A, 1130, and 1135 speciation system (for detailed descriptions of the analytical methods and quality control and quality assurance procedures see [37]). Note that the Tekran system only measures PBM size 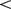
2.5 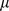
m while removing those 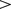
2.5 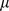
m, which could be an issue for the urban and marine environments which are characterized by large-sized particles [38]. The detection limits for GEM, GOM and PBM were 0.1 ng m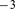, 0.2 pg m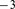, and 0.64 pg m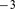, respectively. Since atmospheric Hg concentrations data were available from only one site in the region, we assumed atmospheric Hg concentrations were homogeneous over the entire Adirondacks based on the assumptions that: (1) atmospheric Hg is dominated by a relatively unreactive form (i.e. GEM) in a remote area far from Hg emission sources and with no substantial point emission sources [37], which is likely to be homogeneous in concentration; (2) GEM is the most important deposition form to forested land cover which dominates in the Adirondacks, although GOM and PBM are important atmospheric Hg forms depositing to water surfaces [23]; (3) GEM exhibits high concentration, but low dry deposition velocity. Thus, GEM dry deposition is dominated by its concentration. However, concentrations of GEM are relatively homogeneous and deposition velocity strongly depends on land cover. Thus, the spatial variation of GEM dry deposition is mainly determined by deposition velocity [39]; and (4) HF is located near the geographic center of the Adirondacks at mid elevation and is characterized as largely northern hardwood that is a good representation of the Park. Despite these conditions our analysis is limited by a lack of measured concentrations of Hg forms at various LUCs and elevations across the Park.

We used the Statistic Analysis System (SAS 9.2, SAS Institute Inc., Cary, NC) software to perform the statistical analysis. We used SAS PROC MEANS to calculate the descriptive statistics. We used SAS PROC CORR to analyze the correlations, and PROC GLM with Tukey's method for multiple comparisons. We analyzed and mapped the spatial patterns of atmospheric Hg deposition using Geographical Information System (ESRI ArcGIS 9.3) software.

### General Modeling Scheme

**Figure 3:**
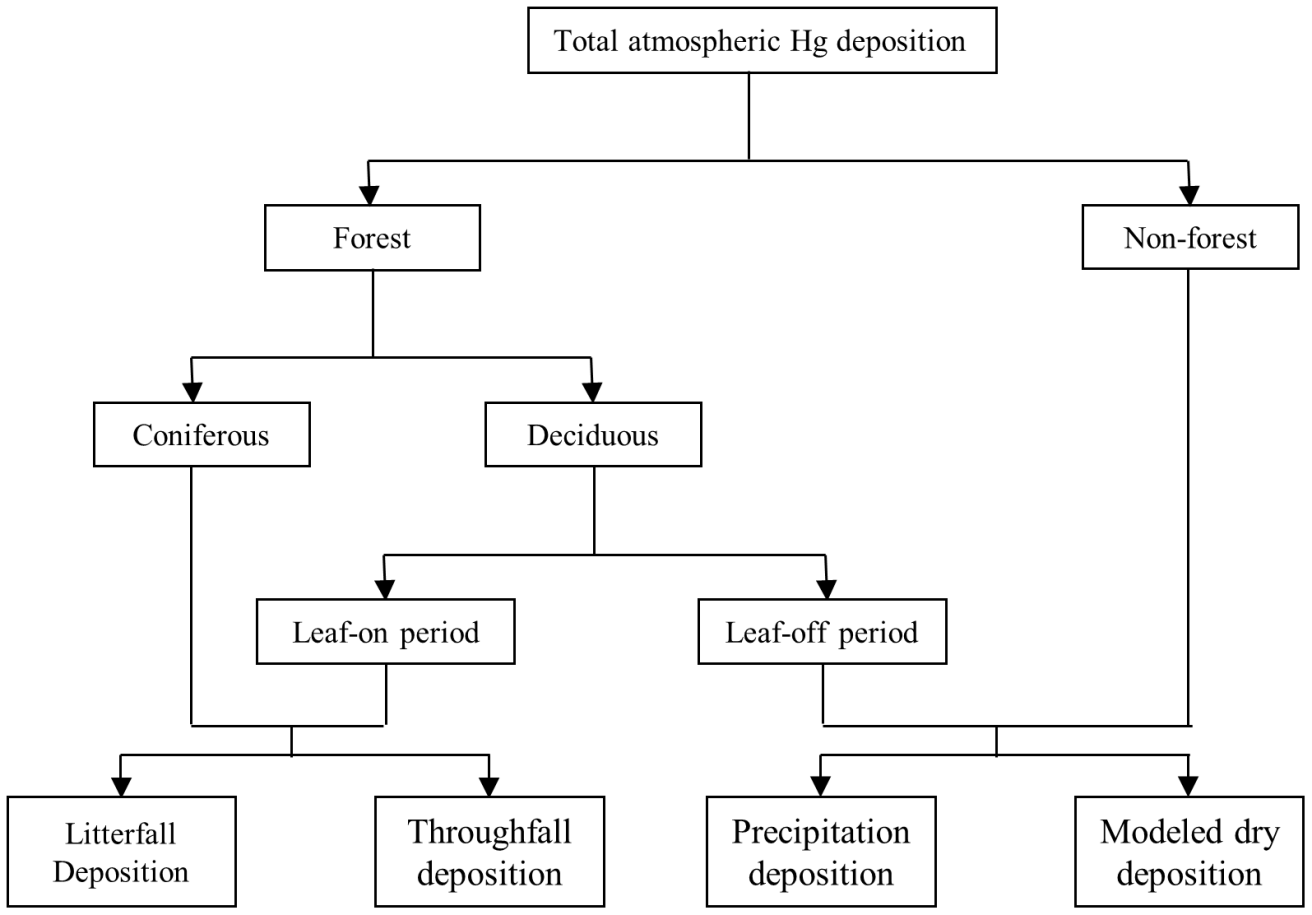
Atmospheric Hg deposition modeling scheme used in this study. Wet Hg deposition is considered as precipitation Hg deposition, while dry Hg deposition is considered as the sum of modeled dry deposition to the non-forested areas and deciduous forest in the leaf-off period, and litterfall and net throughfall (throughfall minus precipitation deposition) Hg deposition to the coniferous forest and deciduous forest in the leaf-on period.

Forests, as the largest component of the Adirondack landscape, play an important role in mediating atmospheric Hg deposition. Forested canopies can substantially enhance atmospheric Hg deposition by providing a large surface area for the direct deposition of PBM [40], through the adsorption and absorption of GOM to the stomata and cuticle [41], by the direct uptake of GEM which is controlled by the stomata and mesophyll resistances [42], [43], and by facilitating the oxidation of GEM to GOM [44]. Therefore, we divided the Adirondack Park into forested and non-forested LUCs, and estimated the atmospheric Hg deposition fluxes using a numerical modeling method (for dry deposition) and experimental data (litterfall, throughfall, precipitation) separately (Figure 3). The evasion of GEM from land surfaces was mapped using a synthesis of experimental data [45]. The net total atmospheric Hg deposition was thus calculated as the total estimated Hg deposition minus land surface GEM evasion.

### Wet Hg Deposition

We estimated wet Hg deposition as the product of volume-weighted Hg concentration in precipitation obtained from MDN and precipitation quantity estimated by PRISM (Parameter-elevation Regressions on Independent Slopes Model; [46]), which spatially depicts precipitation data by considering climatic parameters (temperature, snowfall, growing degree-days, and weather generator parameters) and topographic information from the digital elevation model (DEM). We averaged 2009–2011 data, and interpolated the precipitation Hg concentration and PRISM precipitation quantity data using inverse distance weighted method in ArcGIS.

### Dry Hg Deposition

We estimated dry Hg deposition as the sum of modeled atmospheric dry Hg deposition in the non-forested and deciduous forests during the leaf-off season, and litterfall and net throughfall Hg deposition in the coniferous forests and deciduous forests during the leaf-on period (Figure 3).

#### Dry Deposition

We calculated dry Hg deposition flux (denoted as F, unit: 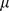
g m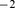 yr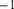) as the product of ambient atmospheric concentration (C, unit: ng m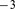 for GEM, and pg m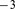 for GOM and PBM) and dry deposition velocity (V_d_, cm s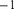) using the Big Leaf model [47]:
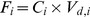
(1)


Gaseous dry deposition velocities for GEM and GOM were calculated using the multiple resistances model (MRM) developed by Wesley and Hicks [26]. This model considers that Hg exchange between the atmosphere and natural surfaces is controlled by a series of resistances which are influenced by meteorological, chemical, physical, and biological conditions. The equation used to calculate dry deposition velocity is expressed as [47]:
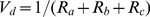
(2)where R_a_ is the aerodynamic resistance, R_b_ is the quasi-laminar sub-layer resistance (which is dependent on the form of Hg), and R_c_ is the canopy resistance [48]. For the deposition to forests, R_c_ is associated with stomatal resistance (R_st_), and non-stomatal resistance which includes in-canopy aerodynamic resistance (R_ac_), soil resistance (R_g_), and cuticle resistance (R_cut_) [48]. We selected the LUCs [49] to correspond with land cover types from the National Land Cover Data (NLCD; [50]) (see Supporting Information). The cuticle and soil resistances used to calculate R_c_ for GEM and GOM were scaled based on estimates for SO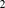 and O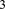, respectively, by:



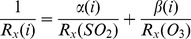
(3)We used values for the two scaling factors 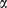 and 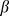 from Zhang et al. [39] (GEM: 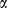 = 0, 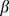  = 0.2; GOM: 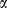 = 10, 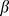  = 10). We also explored the influence of different values of the scaling factors on atmospheric Hg deposition fluxes.

PBM dry deposition velocity was calculated based on Zhang et al. [51] and expressed as:
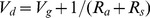
(4)Where V_g_ is the settling velocity resulting from gravity and R_s_ is the surface resistance.

Miller et al. [52] studied the relationships between meteorological conditions and elevation (range: 525–1483 m) at Whiteface Mountain in the Adirondacks. They proposed that the temperature changes by 0.00697°C m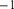, while wind speed follows a logistic model that increases with elevation. Using this model our analysis showed wind speed decreased less than 25% when the elevation decreased from 500 m to 30 m. We adopted their approach to adjust the meteorological conditions when calculating V_d_s at various elevations across the Park.

#### Litterfall Hg Deposition

Studies suggest that Hg translocation from soils to plant canopies is a minor source of Hg in litterfall [53], [54]. However the forest canopy is an active zone of atmospheric Hg deposition and surface GEM re-emission. Generally, Hg concentrations in litterfall are enriched substantially compared to newly grown foliage [55]. The deposition of litterfall is therefore regarded as the difference of atmospheric dry Hg deposition and canopy surface GEM re-emission, but may include some interception of GEM emitted from the soil surface below the canopy [56], [57]. The magnitude of litterfall Hg deposition varies with tree type due to variability in leaf exposure time, ability to adsorb atmospheric Hg, and conditions that influence the conversion between GEM and GOM above and/or near the leaf surface [41], [58]. We used the data from the synoptic study by Risch et al. [59] on litterfall Hg deposition in the eastern U.S., with 14.7 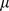
g m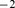 yr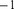 litterfall Hg deposition for deciduous forest, 9.3 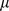
g m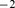 yr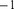 for coniferous forest, and 7.0 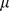
g m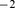 yr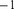 for mixed forest, respectively.

#### Throughfall Hg Deposition

Throughfall is the precipitation that passes through canopies. It exhibits enhanced Hg concentrations due to the leaching of Hg forms from plant tissue surfaces. Rea et al. [55] demonstrated that the increase of Hg concentrations in throughfall was mainly due to wash-off of dry Hg deposition. Throughfall Hg deposition is therefore regarded as the combination of wet Hg deposition and part of the dry deposition to the forest. Demers et al. [36] found that throughfall quantity accounts for around 85% of total precipitation quantity to Adirondack forests. We calculated throughfall Hg deposition from precipitation Hg deposition by adjusting to an enrichment factor K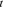 (i.e., the ratio of throughfall/precipitation Hg deposition fluxes). The enrichment factor K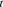 varies by tree type [60], which differ in their ability to capture, adsorb, and mobilize Hg forms. For the deciduous forest, we used an enrichment factor of 1.03 (throughfall Hg deposition 12.0 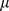
g m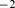 yr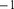, and precipitation Hg deposition 11.6 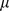
g m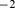 yr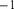) found by Choi et al. [61] for the deciduous forest in the Adirondacks. Witt et al. [60] found an enrichment factor around 1.43 for coniferous forest from pristine sites across the Superior National Forest in northern Minnesota, U.S., which was similar to other studies on throughfall Hg deposition for coniferous forest [40], [44] and was used in this study. For the mixed forest, we used the average of the K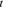 values for deciduous and coniferous forests, with the value of 1.23.

## Results and Discussion

### Atmospheric Hg Concentrations

**Figure 4:**
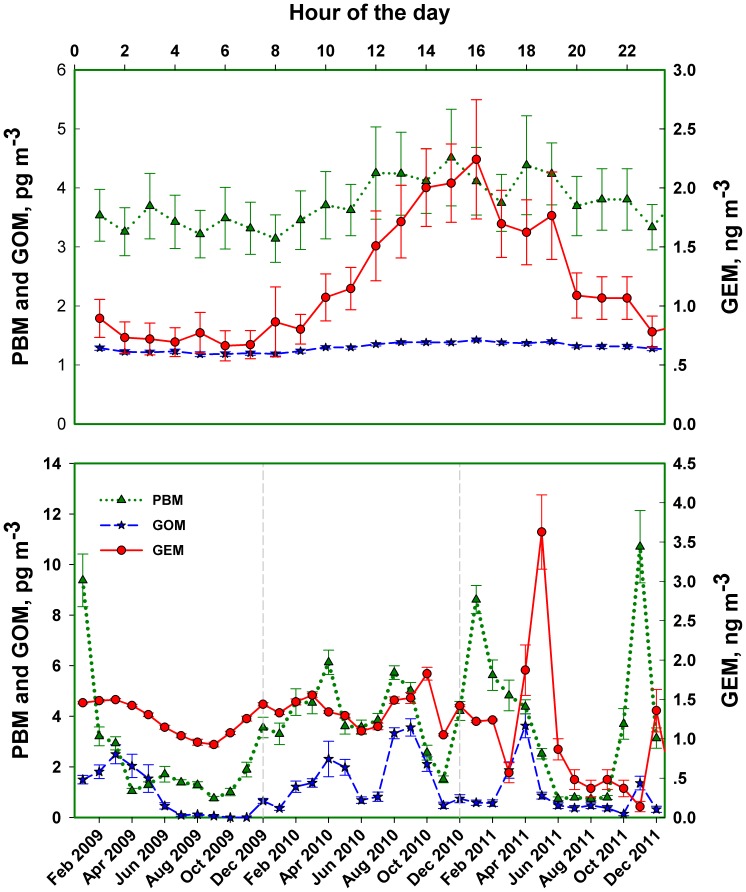
Measured diurnal and seasonal atmospheric Hg concentrations (mean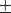 95% confidence value) from 2009–2011 at the Huntington Wildlife Forest in the Adirondack Park. GOM, gaseous oxidized Hg; PBM, particulate bound Hg; GEM, gaseous elemental Hg. Units for GOM and PBM are pg m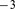, and for GEM is ng m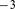.

The average annual concentrations of GEM, GOM, and PBM were 1.30
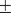
0.31 ng m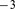, 1.18
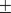
2.22 pg m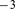, and 3.74
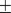
4.90 pg m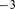, respectively. We used the original data measured by the Tekran monitoring unit when values were below the detection limits to avoid an overestimation of Hg deposition. Concentrations of GEM and PBM during the leaf-off period (GEM: 1.37
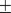
0.23 ng m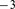; PBM: 5.22
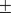
6.58 pg m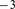) were significantly greater than the leaf-on period (GEM: 1.24
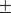
0.35 ng m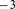; PBM: 2.69
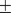
2.74 pg m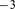), while GOM concentrations were statistically greater during the leaf-on period (1.24
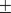
2.48 pg m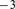, n = 594) than the leaf-off period (1.11
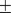
1.80 pg m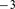, n = 418; Tukey's method, all p-values 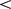
0.0001). Concentrations of all forms of atmospheric Hg showed diurnal patterns peaking in the afternoon (around 15∶00) and the lowest values occurring near midnight (0∶00; Figure 4). Both total atmospheric Hg concentrations and the percentages of oxidized Hg (PBM+GOM) were significantly greater during the leaf-off period than the leaf-on period (Tukey's method, p
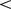
0.0001).

The atmospheric Hg concentrations measured in this study are similar to values reported previously for the HF [37], New England and elsewhere in North America ([62]; and references therein). These results support our assumption that it is reasonable to use the atmospheric Hg concentrations measured at the HF to represent the entire Adirondacks. Meteorological conditions are likely to influence the concentrations of atmospheric Hg by transporting Hg to and from air parcels above the Adirondacks, as well as affecting atmospheric Hg speciation and transformations [38]. However, we did not find a significant relationship between wind speed and atmospheric Hg concentrations. We observed higher PBM and GEM concentrations during the leaf-off period when temperature was lower (Figure 4), which agreed with the slightly negative correlations found between PBM and GEM with surface temperatures on an annual basis.

### Dry Hg Deposition

#### Deposition Velocities

**Figure 5:**
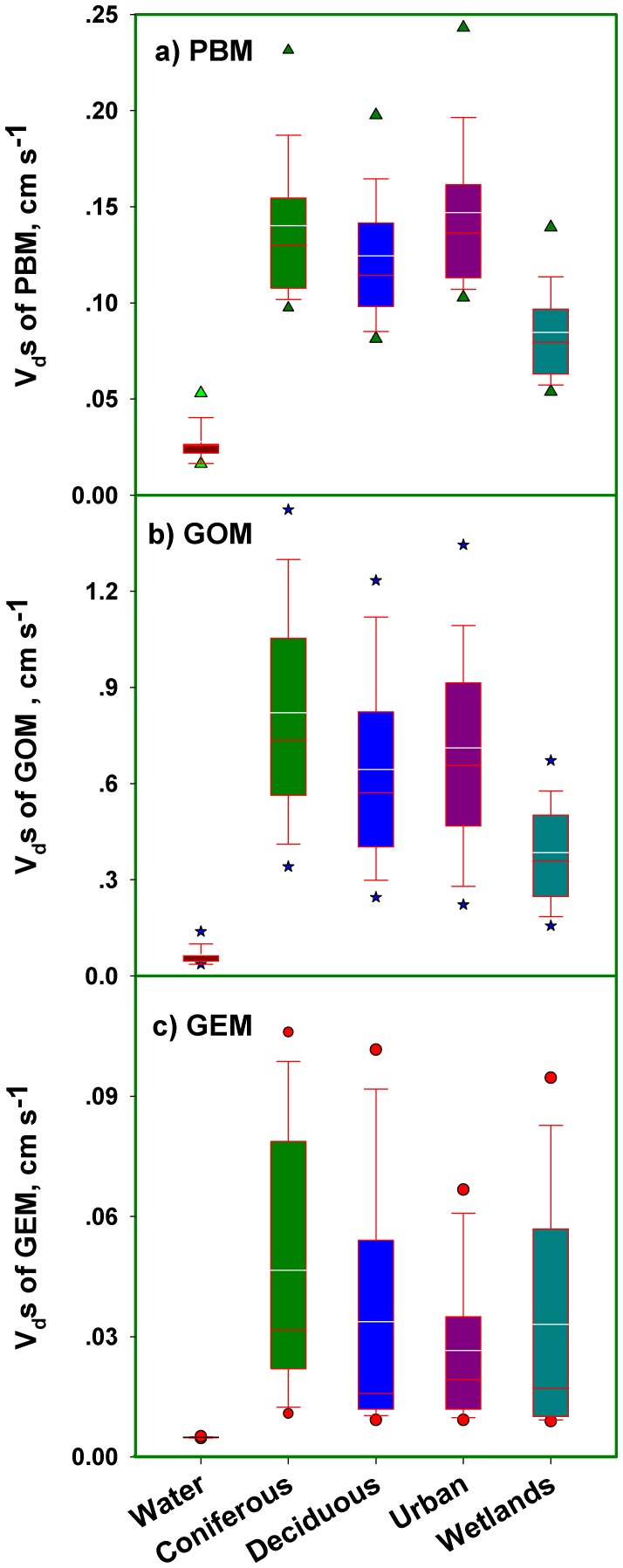
Box plots of deposition velocities (cm s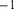) for the major land use categories (LUCs) in the Adirondacks. The upper and lower bars represent the 5th and 95th percentile values, respectively; the bars represent the median (red bars) and mean (white bars) values.

**Figure 6:**
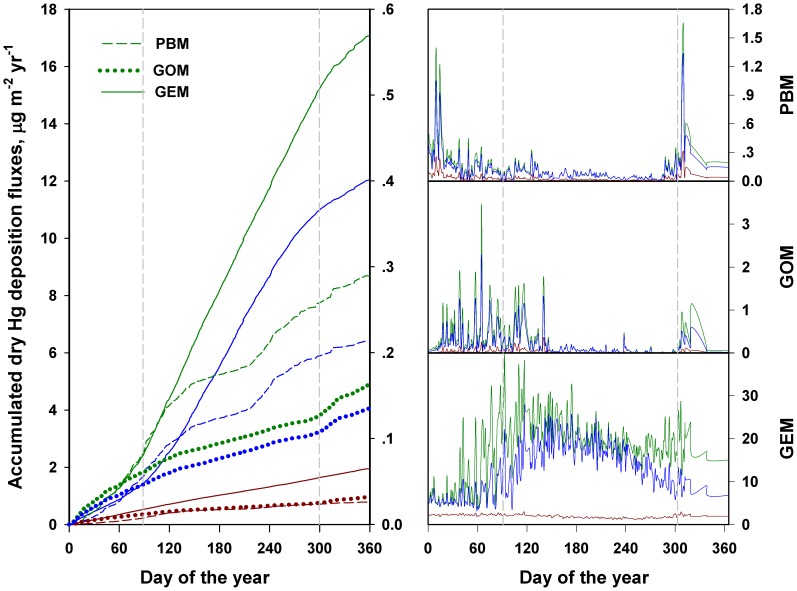
The cumulative and seasonal patterns of annual dry Hg deposition (left panel; GEM left axis, GOM and PBM right axis), and annual dry Hg deposition patterns of the three forms of atmospheric Hg to water, coniferous and deciduous forests in the Adirondacks (right panel).

V_d_s for GOM (average values range from 0.38–0.82 cm s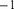 except for water surface) were several fold greater than PBM (0.08–0.15 cm s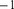), and one or two orders of magnitude greater than GEM (0.02–0.05 cm s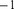). V_d_s for GOM and GEM were greatest for coniferous forests, followed by urban lands, deciduous forests, wetlands, and water surfaces, while the largest values for PBM occurred in urban areas (Figure 5). V_d_s of all atmospheric Hg forms for coniferous forests are greater than deciduous forests, due to the relatively high leaf area indexes of their needle canopies. V_d_s for both PBM and GEM peaked around noon (12∶00), and for GOM peaked around 8∶00 (Figure S1), similar to patterns reported by Zhang et al. [63]. Our modeled V_d_ values for GEM are consistent with the summaries of V_d_s by Zhang et al. [42], while for PBM and GOM they are in the lower range of their summarized data because of the relatively low wind speed in the Adirondacks. V_d_s for GEM for forest LUCs were higher during the leaf-on period, then declined until the end of the year (Figure 6), that may be related to the growth conditions (leaf area index, surface conditions including stomata opening and mesophyll activity) of forest canopies as we observed a correlation between V_d_s for GEM and surface temperature (p
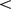
0.0001). V_d_s for PBM and GOM for conifers did not vary significantly throughout the year; while values increased for hardwoods from the beginning of the year to the warm season, then declined to the end of the year. The deposition of PBM and GOM to deciduous forest is more likely related to the forest growth conditions, while the deposition to coniferous forest is additionally influenced by the meteorological variations.

#### Deposition Fluxes

Most of the dry Hg deposition occurred during the leaf-on period (Figure 6). GOM deposition increased from January to April, declined to July, increased to September, then decreased to the end of the year. GEM deposition generally followed the opposite pattern as GOM deposition. The contrasting seasonal patterns of GEM and GOM deposition were in part attributed to the conversion of GEM to GOM, changes in meteorological and forest growth conditions, as well as by the variations of their V_d_ values. The seasonal patterns of PBM and GOM deposition were similar to PBM and GOM concentration patterns, while the seasonal patterns of V_d_ s and fluxes for GEM were also similar. Since F = C* V_d_, the variations of GOM and PBM concentrations are higher than their modeled V_d_ values. Therefore, the dry deposition fluxes (F) of GOM and PBM were largely limited by the species concentrations. However, for GEM, variations of concentrations usually 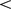
30% (relative standard deviation), but variation of GEM V_d_ s usually ranges from 100 to 150% or higher. Hence, the dry deposition fluxes (F) of GEM is mainly controlled by V_d_.

#### Spatial Patterns of Hg Deposition

**Figure 7:**
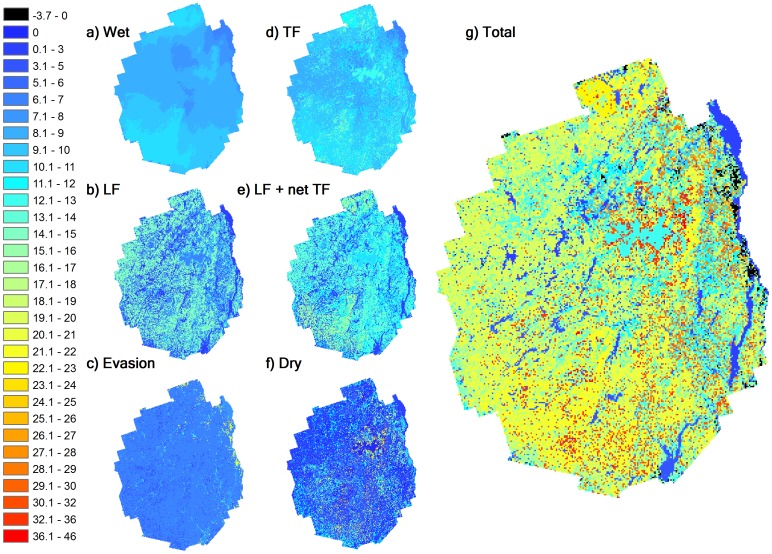
Spatial distribution patterns of atmospheric Hg deposition (unit:
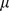
g m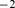 yr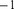 to the Adirondack Park, including: a) wet deposition, b) litterfall deposition (LF), c) surface GEM evasion, d) throughfall deposition (TF), e) the sum of LF and net TF (TF - wet deposition), f) the modeled dry deposition (sum of GEM, GOM and PBM deposition), and g) the total net Hg deposition (wet+dry - evasion).

The average total net atmospheric Hg deposition to the Adirondacks from 2009–2011 was 17.4 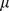
g m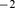 yr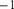, with a range of −3.7–46.0 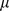
g m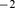 yr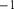 (Figure 7). The total Hg deposition, dry and wet Hg deposition, and GEM evasion were 580, 370, 210 and 170 kg yr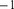, respectively for the entire Adirondack Park (2.4 million ha). Dry Hg deposition mainly occurred as GEM deposition (97.5%), which is similar to the findings of a recent study by Huang et al. [10]. The deciduous forest lands in the Adirondacks received the greatest net atmospheric Hg deposition (224 kg yr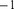; area: 1.12 million ha), followed by mixed forest (70 kg yr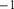; 0.25 million ha), coniferous forest (64 kg yr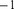; 0.46 million ha), wetlands (46 kg yr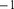; 0.28 million ha), water (5 kg yr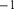; 0.15 million ha), and urban areas (3 kg yr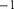; 0.05 million ha).

Wet Hg deposition, which ranged from 6–11 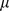
g m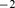 yr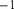, did not vary substantially throughout the Adirondacks, though it was relatively lower to the water surfaces in the northeast and higher in locations that coincide with higher elevations diagonally from the southwest through the center of the Park (Figure 7). The spatial pattern of both litterfall and throughfall Hg deposition were mainly governed by the distribution of forest types, while there was large spatial variation for the modeled dry Hg deposition (Figure 7). There were scattered areas in the east which had atmospheric Hg deposition greater than 30 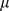
g m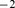 yr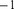, while the southwestern and the northern areas received relatively high Hg deposition ranging from 25–30 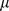
g m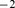 yr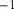. The spatial patterns of atmospheric Hg deposition were similar to the distribution of land cover types, especially forests, in the Adirondacks.

**Figure 8:**
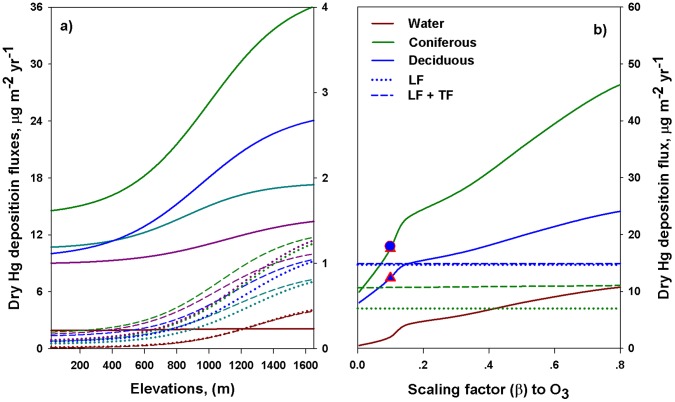
Plot a): Relationships of modeled Hg dry deposition fluxes with elevation (GEM left axis, GOM and PBM right axis). The logistic equations used are shown in SI Table A. Plot b): The effect of the scaling factor (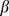) on the modeled dry Hg deposition, and the comparisons with field data (solid lines: modeled dry deposition; while the dotted, dashed, and dash-dotted lines: the field data of litterfall Hg deposition and the sum of litterfall (LF) and net throughfall (TF) Hg deposition, respectively). Note that the net throughfall Hg for deciduous forest is small, with the values for LF Hg deposition and the sum of LF and TF Hg deposition as 14.7 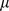
g m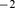 yr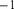 and 14.9 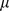
g m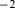 yr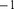, respectively. In this study, we used a value of 0.1, and the corresponding modeled dry Hg deposition to coniferous (dark green triangle symbol) and deciduous (blue triangle symbol) forests were: 17.5 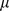
g m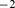 yr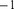 and 12.4 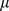
g m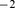 yr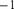, respectively. The blue circle that nearly supersedes the dark green triangle represents the modeled net dry Hg deposition to deciduous forest (17.9 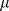
g m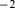 yr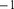).

The net throughfall Hg deposition (throughfall minus wet Hg deposition) was small compared to litterfall deposition (Figure 8b) for the deciduous forest [36]. Note that field observations using this method likely underestimate true dry Hg deposition (the dry deposition of all forms of atmospheric Hg, including those below the detection limit and not measured) as there are likely re-emissions of Hg deposited to the canopy and stem flow which is not quantified by net throughfall plus litterfall Hg data.

The modeled dry deposition to the forest, especially the coniferous forest, was much greater than experimental observations (Figure 8b). The coniferous forest received higher dry Hg deposition than deciduous forest. However, the net total Hg deposition estimated for the coniferous forest (12.6 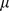
g m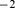 yr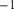) was lower than the deciduous forest (20.0 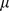
g m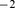 yr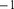), which agrees with field results which found lower litterfall Hg deposition in the coniferous forest [59]. This apparent disparity between dry deposition and net Hg deposition can be explained by the fact that Hg concentrations in deciduous foliage and litterfall mass were greater than for conifers, though more Hg might be retained by coniferous forest [57].

### Model Evaluation

#### Multiple Resistances Analysis

The three serial resistances (R_a_, R_b_, and R_c_) considered in calculating dry Hg deposition velocity were equally important in the calculation of V_d_s of GOM for the forest LUCs, while R_c_ was the dominant component of V_d_s for GEM (Figure S2). The GEM deposition velocity is largely controlled by processes on the leaf surfaces. Examination of the components contributing to R_c_ from stomatal resistance (R_st_), ground resistance (R_g_), and cuticle resistance (R_cut_), showed quite different patterns for coniferous and deciduous forests (Figure S3). Cuticle resistance was equally important throughout the entire year for coniferous forest, while it was more important during the leaf-on period than the leaf-off period for the deciduous forest. The ground resistance was as important as cuticle resistance for the coniferous and deciduous forest during the leaf-on period, while it was much more important than cuticle resistance for the deciduous forest during the leaf-off period.

#### Dry Deposition Evaluation

The calculated dry Hg deposition fluxes followed a similar logistic pattern as wind speed with elevation (Figure 8a; model equations in Table A in File S1), which confirmed that wind speed is the dominant parameter driving dry Hg deposition velocities. The estimates of Hg deposition increased from several fold to an order of magnitude from the lowest to the highest elevation across the Adirondacks. The fraction of GOM and PBM to the total dry Hg deposition also increased with increases in elevation.

The most difficult and problematic aspect of estimating atmospheric Hg deposition is quantifying the contribution of GEM deposition to the total Hg deposition. Recent modeling studies have addressed the importance of GEM deposition, especially to forest canopies [23], [39]. However, understanding of atmospheric Hg deposition to canopies is limited. Our current method to calculate V_d_s for Hg deposition is based upon scaling to a well-studied modeling method used for SO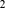 (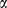), and O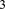 (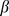). We conducted a sensitivity analysis of 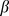 values for GEM to examine the relative contribution of GEM deposition to the total atmospheric Hg deposition. The results showed that GEM deposition was positively correlated with 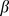 values (Figure 8b). The sensitivity analysis of the scaling factors has important implications in guiding future modeling studies in selecting optimum scaling factors based on field measured dry deposition data.

#### Leaf-on vs. Leaf-off Periods

One of the main considerations of the modeling scheme used in this study is the separation of the leaf-on and leaf-off periods for the deciduous forest, and the use of litterfall and throughfall Hg deposition data from the literature instead of the modeled dry Hg deposition. Our modeled PBM and GOM dry deposition was greater during the leaf-off period, while GEM deposition was greater in the leaf-on period. The modeled dry Hg deposition during the leaf-on period for the deciduous forest was 8.9 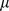
g m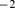 yr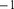 at 30 m elevation, 9.6 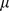
g m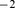 yr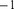 at 500 m, and 19.4 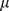
g m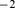 yr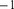 at 1630 m, which are within the range of the litterfall deposition values reported by Risch et al. [59] (14.7 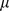
g m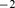 yr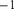; the site elevations are around 500 m), and Bushey et al. [57] (for the HF, 16.4–17.9 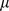
g m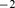 yr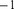).

#### Model Intercomparison

We compared estimates of atmospheric Hg deposition with the results from Miller et al. [23], CMAQ-2005 [33], and Zhang et al. [63] for the Adirondacks. Miller et al. [23] estimated the total Hg deposition as the sum of precipitation Hg deposition, GEM assimilation by vegetation, dry deposition of GOM and PBM, and cloud-droplet interception, with the resulting deposition flux of 25.7 (range: 1.3–37.6) 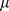
g m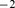 yr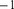 and total Hg deposition of 610 kg yr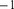 for the Adirondack Park. Our modeled Hg deposition are lower than values estimated by Miller et al. [23], which is probably due to the different methods used in estimating GEM dry deposition. Miller et al. [23] used foliage Hg accumulation and leaf litterfall rate to estimate GEM dry deposition, which may not be able to reflect the diurnal patterns of GEM deposition fluxes and the elevational effect. The spatial distribution patterns of the atmospheric Hg deposition in our study and Miller et al. [23] were similar.

CMAQ-2005 [33] estimated total Hg deposition as the sum of modeled wet and dry Hg deposition. The atmospheric Hg concentrations used were estimated from GEM emission inventories and their subsequent dispersion, transport and reactions. Dry deposition was estimated using similar methods with this study. The modeled Hg deposition by CMAQ-2005 for the Adirondacks [33] was 27.0 (range: 6.7–51.7) 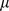
g m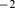 yr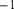, with a total amount of 640 kg yr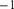 for the Adirondacks. The estimated dry deposition of GEM modeled by CMAQ-2005 [33], which was not considered in the previous version [19], was similar to our results. However, the dry deposition of GOM modeled by CMAQ-2005 [33] was equally important with GEM, which resulted in a higher estimation of total Hg deposition than we observed. Note that the measurements of GOM and PBM concentrations by the Tekran might be lower than actual values (according to [64], 41% of the actual GOM concentrations). As a result, GOM dry deposition might be higher than our estimates (based on [64], possibly two times higher), although this again appears to be a small fraction (3%) of the total Hg deposition. On the other hand, CMAQ may overestimate the deposition of reactive Hg (GOM+PBM) due to potential errors in Hg emission inventories and speciation as well as the impacts of in-plume Hg reduction [25].

Zhang et al. [63] used a method similar to our study to estimate dry Hg deposition to 19 monitoring locations in Eastern and Central North America, which included our monitoring site at HF. The average PBM, GOM and GEM deposition fluxes at HF from Zhang et al. [63] were 0.15 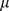
g m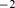 yr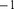, 0.32 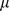
g m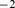 yr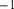, and 16.0 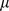
g m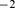 yr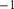, respectively. Oxidized Hg (PBM 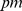 GOM) was approximately 20% times higher than estimated in our study, however for GEM the deposition value was 5% times lower. Overall the total dry Hg deposition fluxes were in good agreement (16.5 vs 17.2 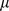
g m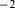 yr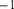).

### Conclusion

In this study, we estimated atmospheric Hg deposition using a new scheme that combined numerical modeling and limited experimental data. The average atmospheric Hg deposition to the Adirondacks was estimated as 17.4 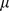
g m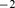 yr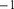, with a range of −3.7–46.0 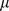
g m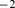 yr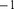. The spatial pattern of atmospheric Hg deposition showed a large variation across the Adirondacks, with scattered areas in the eastern Adirondacks which had total Hg deposition greater than 30 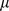
g m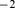 yr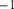, while the southwestern and the north areas received Hg deposition ranging from 25–30 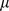
g m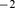 yr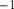.

Although atmospheric Hg deposition to the Adirondacks is modest, it exhibits considerable spatial variation over the Park. There are two overarching patterns that drive Hg deposition to the Adirondacks. Firstly, Hg deposition is higher in areas with forest land cover. Forest canopy provides a large surface area which facilitates the removal of atmospheric Hg forms. The deposited Hg is subsequently transported to the land in the processes of litterfall and throughfall deposition. The estimate of atmospheric Hg deposition in this study, together with previous modeling [23], [63] and field monitoring of Hg deposition in forests [57], [59] confirms this pattern. Secondly, Hg deposition is likely to be higher in greater elevation areas. There are a few field studies [65], [66] as well as a modeling study [23]) that consider the effect of elevation on Hg deposition. Hg deposition velocities increased with increasing elevation due to increases in wind speed, although the effect may be offset by the relatively lower temperature at these sites. At higher elevations, the effects of atmospheric Hg deposition may be exacerbated due to the shallower soils and more sensitive landscape characteristics [4], [67], [68].

## Supporting Information

Figure S1 **Diurnal patterns atmospheric Hg deposition velocities (mean ±95% confidence value)) for coniferous (dark green lines) and deciduous (blue lines) forest.**
(TIF)

Figure S2 **The contribution patterns of the serial resistances (Ra, Rb, Rc; average values) in calculating atmospheric Hg deposition velocities to coniferous forest, deciduous forests and water.**
(TIF)

Figure S3 **The annual contribution patterns of the components in calculating the canopy resistance (Rc) of atmospheric Hg deposition velocities to coniferous (plot a) and deciduous forest (plot b).**
(TIF)

File S1 (DOCX)
