# Supplementary figures and images for: Modeling and Mapping of Atmospheric Mercury Deposition in Adirondack Park, New York
Source: PLoS One. 2013 Mar 25;8(3):e59322. doi: 10.1371/journal.pone.0059322 (PMC3607617; doi:10.1371/journal.pone.0059322)

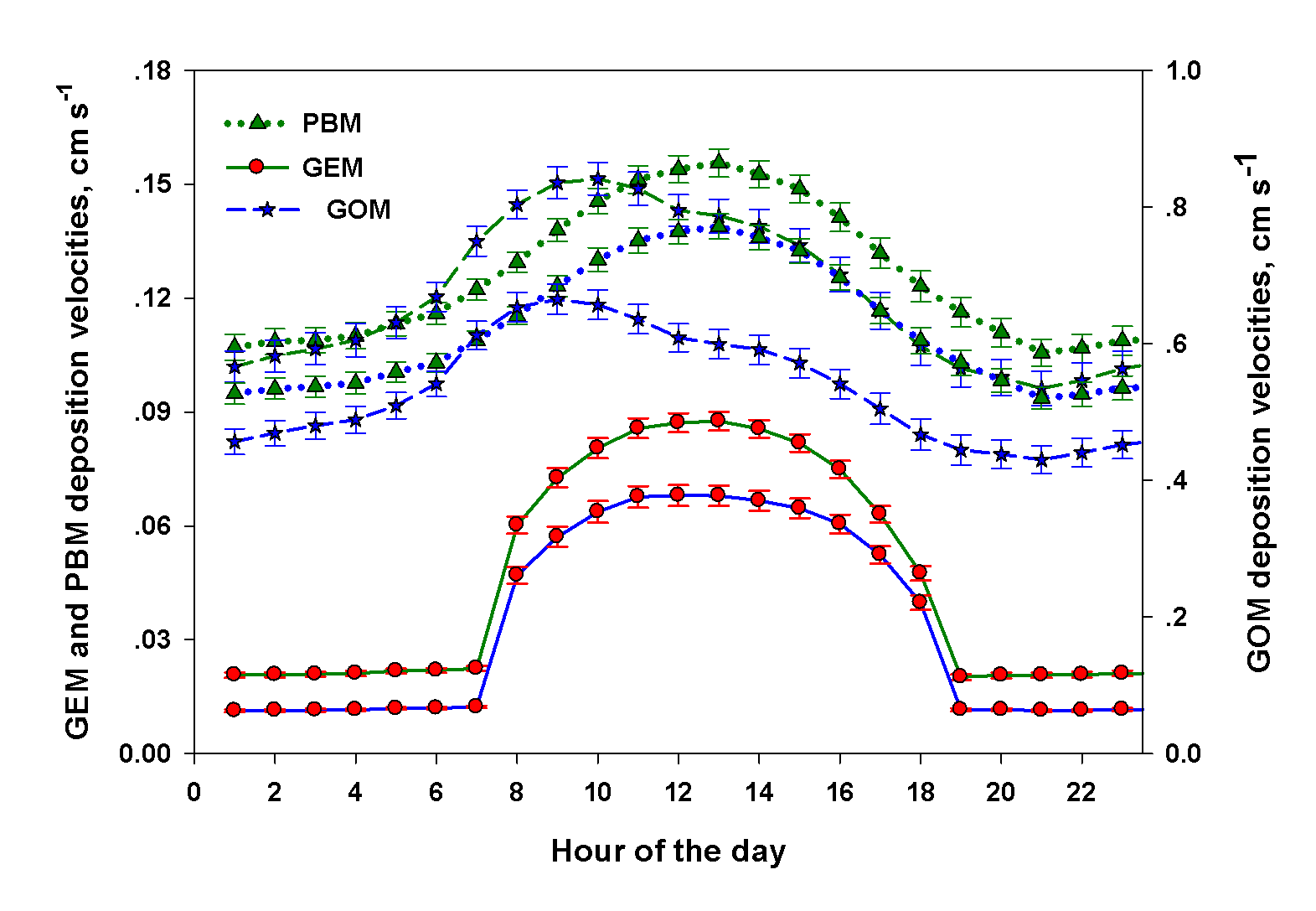

Supplement: Figure S1 — Diurnal patterns atmospheric Hg deposition velocities (mean ±95% confidence value)) for coniferous (dark green lines) and deciduous (blue lines) forest. (TIF) [file pone.0059322.s001.tif]

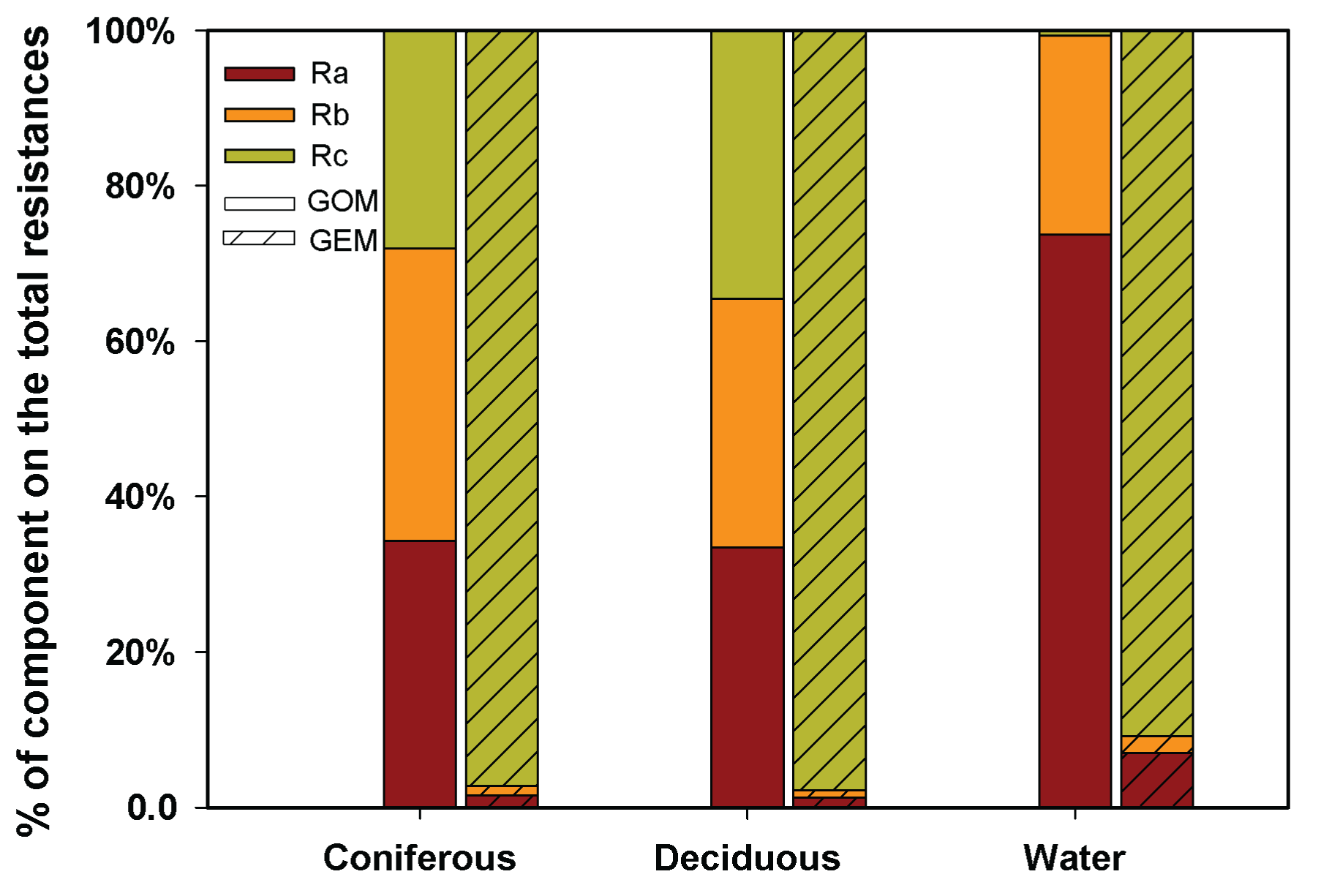

Supplement: Figure S2 — The contribution patterns of the serial resistances (Ra, Rb, Rc; average values) in calculating atmospheric Hg deposition velocities to coniferous forest, deciduous forests and water. (TIF) [file pone.0059322.s002.tif]

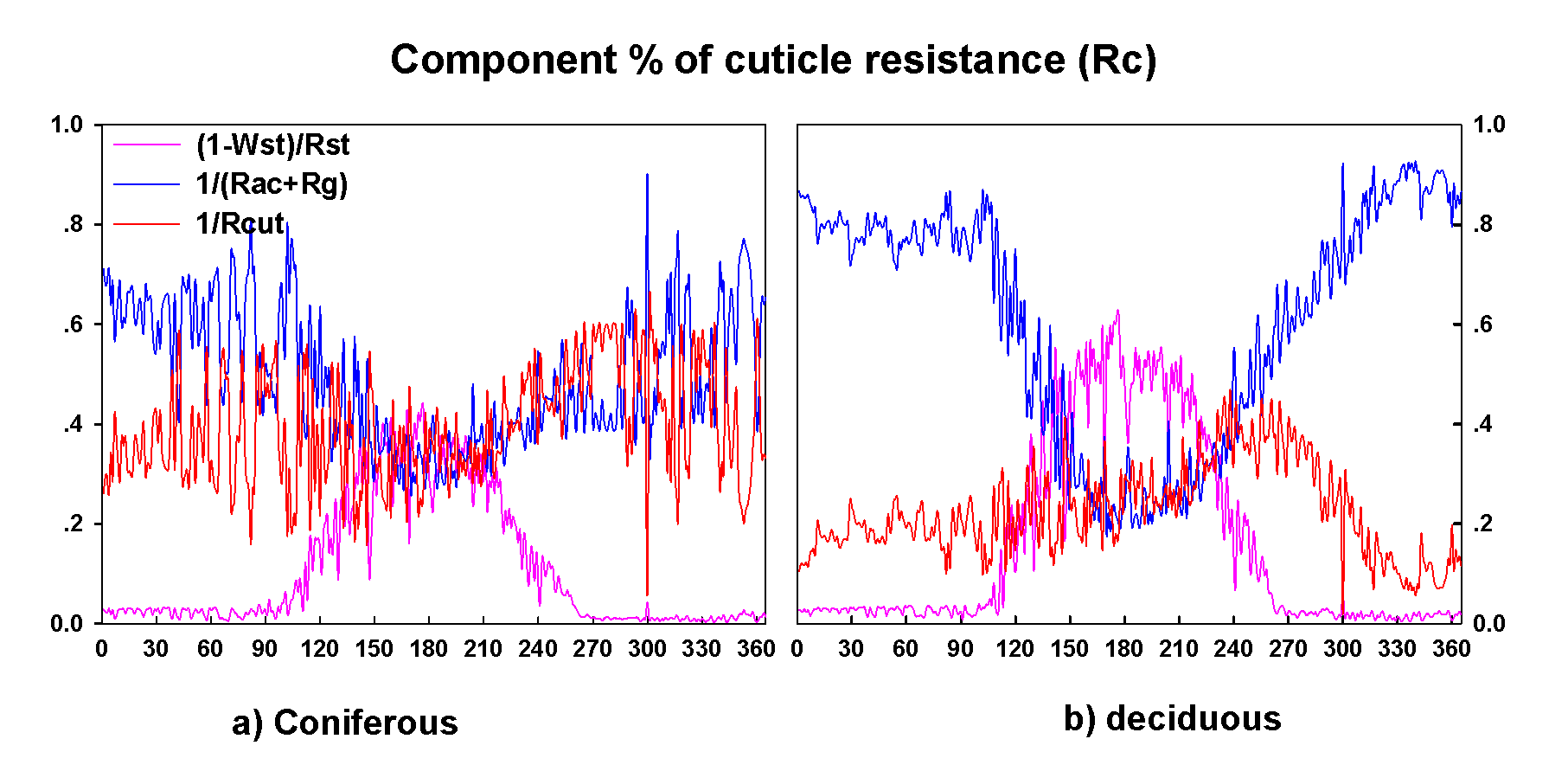

Supplement: Figure S3 — The annual contribution patterns of the components in calculating the canopy resistance (Rc) of atmospheric Hg deposition velocities to coniferous (plot a) and deciduous forest (plot b). (TIF) [file pone.0059322.s003.tif]
