# Supplementary material for: Modeling and Mapping of Atmospheric Mercury Deposition in Adirondack Park, New York
Source: PLoS One. 2013 Mar 25;8(3):e59322. doi: 10.1371/journal.pone.0059322 (PMC3607617; doi:10.1371/journal.pone.0059322)
Supplement: File S1 — (DOCX) [file pone.0059322.s004.docx]

**Modeling and mapping of atmospheric mercury deposition in Adirondack Park, New York**

**Xue Yu^1,*^, Charles T. Driscoll^1^, Jiaoyan Huang^2^, Thomas M. Holsen^3^, and Bradley D. Blackwell^1^**

^1^ Department of Civil and Environmental Engineering, Syracuse University, Syracuse, NY 13244

**Supporting Information**

**(3 pages, 1 table, 3 figures)**

**Corresponding LUCs developed in Zhang et al. (2002) to the land cover types from NLCD**

We matched the land use denoting codes used in model developed by Zhang et al. (2002) to the land use cover types with the land cover types from the National Land Cover Data (NCLD) based on the principal of similarity. We also grouped several types of land cover from NCLD, which have very small area coverage in the Adirondacks, into one bigger representing type. We selected LUC #3 from Zhang et al. (2002) as open water (grid code # 11 ), LUC # 21 as the developed lands (Grid code # 21-24, 31), LUC #7 as deciduous forest (grid code # 41), LUC # 4 as evergreen forest (grid code # 42), LUC # 25 as mixed forest (grid code # 43), and LUC #23 as wetlands (grid code # 52, 71, 81, 82, 90, 95).

**Reference:**

Zhang L, Moran M, Makar P, Brook J, Gong S (2002) Modelling gaseous dry deposition in AURAMS: a unified regional air-quality modelling system. Atmospheric Environment 36: 537-560.

**Figure Legends**

Figure S1. Diurnal patterns atmospheric Hg deposition velocities (mean ± 95% confidence value)) for coniferous (dark green lines) and deciduous (blue lines) forest.

Figure S2. The contribution patterns of the serial resistances (R_a_, R_b_, R_c_; average values) in calculating atmospheric Hg deposition velocities to coniferous forest, deciduous forests and water.

Figure S3. The annual contribution patterns of the components in calculating the canopy resistance (R_c_) of atmospheric Hg deposition velocities to coniferous (plot a) and deciduous forest (plot b).

Table A in File S1. Modeled logistic growth equations of atmospheric Hg deposition flux (y, μg m^-2^ yr^-1^) as a function of elevation (x, m) for each land use categories

| Species | No. | Name | Equation |
| --- | --- | --- | --- |
| GEM | LUC 3 | Water | y=1.8927+0.2115/(1+exp(-(x-730.3987)/235.6775)) |
|  | LUC 4 | Coniferous forest | y=13.9842+23.9444/(1+exp(-(x-1001.6004)/262.0736)) |
|  | LUC 7 | Deciduous forest | y=9.5216+15.6418/(1+exp(-(x-950.02)/271.5267)) |
|  | LUC 21 | Urban | y=8.8890+5.1593/(1+exp(-(x-1098.4524)/282.0117)) |
|  | LUC 23 | Wetland | y=10.5053+7.0025/(1+exp(-(x-856.7495)/232.6442)) |
|  | LUC 25 | Mixed forest | y=13.8472+22.1474/(1+exp(-(x-991.8694)/261.6689)) |
| GOM | LUC 3 | Water | y=0.0074+0.5179/(1+exp(-(x-1275.0311)/234.9505)) |
|  | LUC 4 | Coniferous forest | y=0.1776+1.2268/(1+exp(-(x-1060.7577)/243.3295)) |
|  | LUC 7 | Deciduous forest | y=0.1403+1.0122/(1+exp(-(x-11226.8316)/244.9482)) |
|  | LUC 21 | Urban | y=0.1552+1.0350/(1+exp(-(x+1039.4974)/247.6679)) |
|  | LUC 23 | Wetland | y=0.0762+0.8392/(1+exp(-(x-1140.0212)/260.1457)) |
|  | LUC 25 | Mixed forest | y=0.1592+1.1458/(1+exp(-(x-1092.5013)/244.6109)) |
| PBM | LUC 3 | Water | y=0.0132+0.5451/(1+exp(-(x-1306.2746)/239.8061)) |
|  | LUC 4 | Coniferous forest | y=0.0843+1.3684/(1+exp(-(x-1225.4879)/257.1027)) |
|  | LUC 7 | Deciduous forest | y=0.0739+1.1268/(1+exp(-(x-1227.2206)/252.9323)) |
|  | LUC 21 | Urban | y=0.0883+1.3962/(1+exp(-(x-1214.8897)/256.1466)) |
|  | LUC 23 | Wetland | y=0.0522+0.8848/(1+exp(-(x-1248.204)/256.2931)) |
|  | LUC 25 | Mixed forest | y=0.0775+1.219/(1+exp(-(x-1223.9013)/254.5275)) |
| Deciduous forest in the leaf-off period | | | |
| GEM | | | y=5.048/(1+exp(-(x-499.5449)/418.8136) |
| GOM | | | y=0.0458+0.368/(1+exp(-(x-1147.5)/262.9804)) |
| PBM | | | y=0.0358+0.6746/(1+exp(-(x-1227.5610)/256.8468)) |
